# Numerical and Experimental Investigation into LWIR Transmission Performance of Complementary Silicon Subwavelength Antireflection Grating (SWARG) Structures

**DOI:** 10.1038/s41598-019-41107-2

**Published:** 2019-03-18

**Authors:** Ramazan Cetin, Tayfun Akin

**Affiliations:** 1 METU MEMS Centre, Middle East Technical University, Ankara, 06510 Turkey; 2 Electrical and Electronics Eng. Department, Middle East Technical University, Ankara, 06800 Turkey

## Abstract

This paper presents a detailed comparison between the long wave infrared (LWIR) transmission performances of binary, silicon based, structurally complementary pillar and groove type antireflective gratings that can be used for wafer level vacuum packaging (WLVP) of uncooled microbolometer detectors. Both pillar and groove type gratings are designed with various topological configurations changing in various period sizes (Λ) from 1.0 μm to 2.0 μm, various heights/depths (h) from 0.8 μm to 1.8 μm, and various pillar/groove width-to-period (w/Λ) ratios from 0.6 to 1.0. The transmission performance of gratings is simulated with a hybrid simulation technique based on the modification of the reflection term within the Fresnel transmission equation, which combines both numerical and analytical approaches in a unique way for the first time in literature. Simulation results are experimentally verified with 19 different fabricated structures where a spectral agreement is achieved with an absolute root-mean-square (RMS) error less than 5.4% within the subwavelength (SW) regime, proving the effectiveness of the proposed hybrid technique. These results show first time in the literature that both pillar and groove type silicon based gratings present similar spectral IR transmission characteristics, and they are also structurally complementary when optimum configurations are employed to maximize the transmission.

## Introduction

Advances in uncooled infrared (IR) technology have been decreasing the cost of long wave infrared (LWIR) detector arrays, allowing their use in wide range of applications; however, the cost of their vacuum packaging using ceramic or metal is still a bottleneck for their use in low cost applications, such as security, autonomous driving, IoT, and consumer applications. A solution for low cost vacuum packaging is to use wafer level vacuum packaging (WLVP) techniques, i.e., attaching a silicon cap wafer on the detector wafer to achieve a vacuum condition for the microbolometer detector in the die level, in which case the IR transmission performance of the silicon cap wafer becomes a critical issue^1–3^. A double side polished (DSP) mono-crystalline silicon (Si) based cap wafer typically transmits around 50% within the LWIR (8–12 µm) wavelength range; nevertheless, this can be increased above 85% with antireflective (AR) coating of stacked layers on both sides of the cap wafer^4,5^. However, the use of AR stack layers is not trivial, requiring a costly, complex technology development for achieving a high transmission performance in the wide bandwidth while avoiding delamination and performance degradation issues especially at high temperatures necessary for WLVP steps^6,7^. In order to reduce temperature exposures below 300 °C, gold-based eutectic bonding techniques in WLVP are needed; however, the use of gold is not only expensive but also a contaminant approach for CMOS foundries.

An alternative to AR coating of stacked layers for increasing the transmission is the utilization of subwavelength (SW) antireflective gratings (SWARGs), which is achieved by patterning pillar-like (also referred as moth-eye) or groove-like (also referred as inverse moth-eye) structures with a high enough spatial frequency through micromachining on the surface of the substrate^6,8^. Inspired by biomimetics, these grating based AR structures eliminate the need for AR coating of stacked layers by providing the substrate itself, which is the silicon cap wafer within the scope, with antireflective characteristics. Although there are many studies examining these structures, there is still no study in the literature with conclusive results comparing relative performances of both grooves and pillars one to another.

The most widely used approaches for simulation and modelling of SW grating structures are the effective medium approach (EMA) and the rigorous couple wave analysis (RCWA) algorithm^6,8–11^. Nonetheless, this study presents a novel hybrid simulation technique combining the efficient 3D modelling capability of packaged programs, such as HFSS or Lumerical with the incoherent analysis capability of Fresnel transmission equations to handle the electrically thick nature of the substrate. The proposed method is based on modifying the reflection term of the Fresnel equation through the 3D electromagnetic (EM) analysis of the gratings. However, the proposed method is only valid within SW regime, and thus it can be seen as an alternative approach for RCWA and EMA techniques while handling EM modelling of subwavelength structures. Still, it is observed that the proposed hybrid simulation technique presents conclusive results while predicting the spectral IR transmission of both grooves and pillars. The spectral responses of both structures are examined through various topological configurations changing in period sizes (Λ), heights/depths (h), and width-to-period (w/Λ) ratios, for all of which an excellent spectral agreement between the measurements and simulations is obtained. This technique is used to demonstrate first time in the literature that binary type pillars and grooves present similar spectral IR transmission performance when optimum configurations are employed to maximize the transmission, and they also turn out to be structurally complementary, which is also verified through measurements and explained through an analytical model.

## Results

### Design, Simulation and Optimization

**Figure 1:**
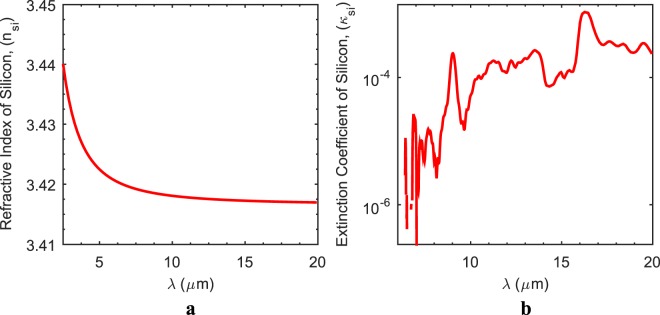
Optical constants of Czochralski (CZ) type Si wafer. The refractive index (n_si_) is extracted directly from the literature data (**a**), while the extinction coefficient (κ_si_) is recalculated through IR transmission measurement of a plain DSP wafer (**b**).

Design of pillar and groove type antireflective gratings is achieved with utilization of the quarter wave index matching (QWIM) technique, in the same way that it is utilized for the design of the AR coating of stacked layers, where the silicon transmission is enhanced with the minimization of reflection through destructive interference^6,10,12^. However, unlike the stacked layer case where a bulk refractive index is looked for, an effective refractive index (n_eff_) is searched for index-matching of silicon (n_si_) to the air (n_air_), such as n_eff_ = (n_air_ × n_si_)^0.5^, with the employment of SWARGs since the volume composed of SW gratings is considered as a composite layer, which is partially air and partially silicon. Within the scope of this study, optical constants of silicon, i.e., the refractive index (n_si_) and the extinction coefficient (κ_si_), are extracted from the literature^13^. As known, there are mainly two types of Si wafers in the market, namely the Float Zone (FZ) and the Czochralski (CZ) type wafers, which mainly differ in their production techniques and LWIR range extinction coefficients^14^. Although an FZ type wafer has a higher LWIR transmission since it does not have an absorption peak at 9 µm in contrast to a CZ type, it is more expensive than the CZ type due to its production technique; thus, the CZ type wafer is employed within the scope of this study due to cost issues^14,15^. The extinction coefficient of CZ type wafer is recalculated from the IR transmission of a plain DSP wafer instead of using the literature data in order to have a better matching between simulations and measurements^6^. Figure 1 shows the optical constants of CZ type wafer employed within the scope.

**Figure 2:**
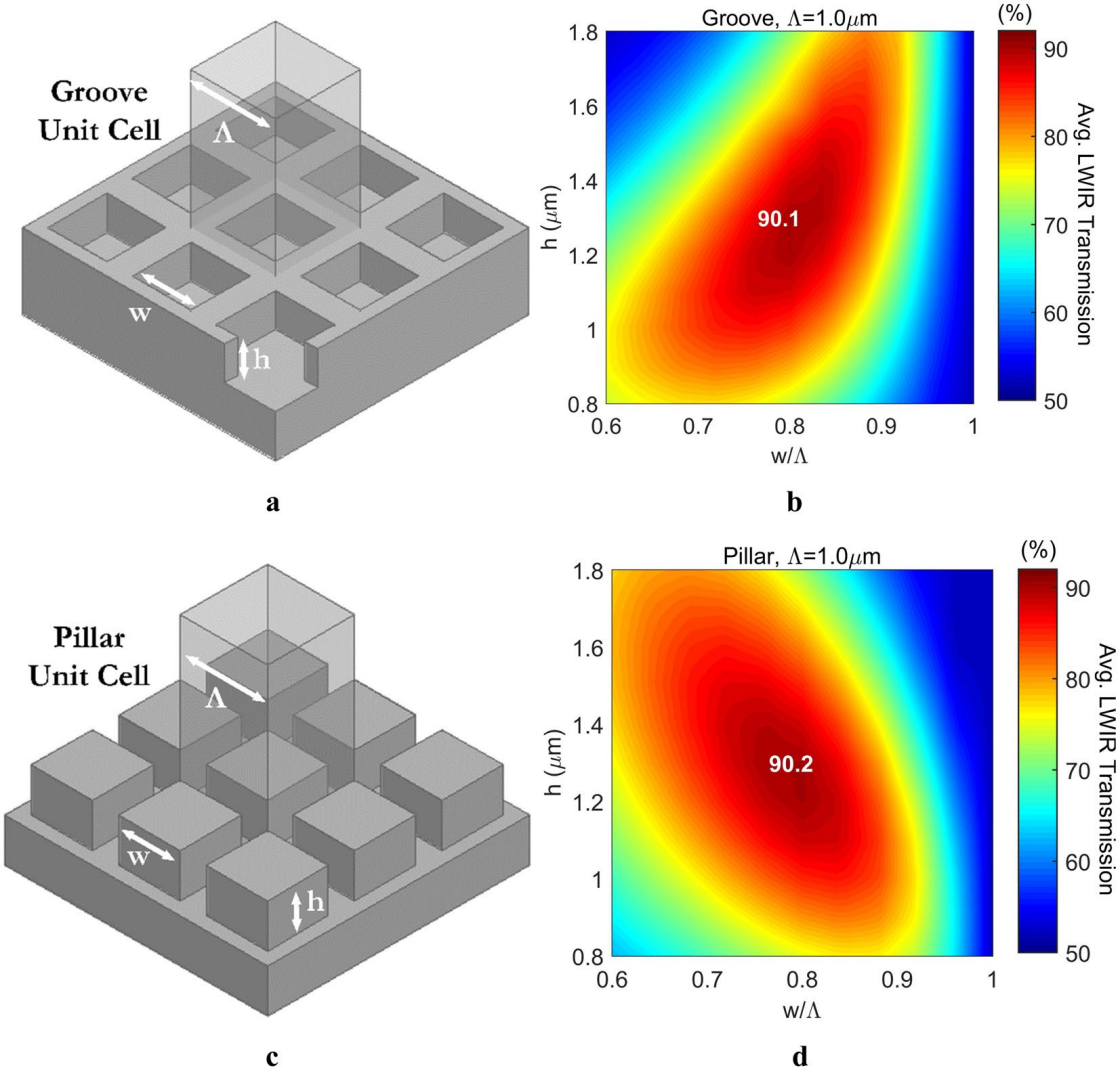
3D schematics illustrating the SWARG unit cells and 2D optimization results for maximum LWIR transmission. Topological parameters and the 3D patterning for both groove (**a**) and pillar **(c)** type SWARGs are illustrated. 2D optimization results show that both grooves (**b**) and pillars (**d**) not only present a similar maximum LWIR transmission around 90% but also the same optimum dimensions for maximum transmission, assuming double side patterning with a period of Λ = 1.0 µm.

Figure 2a,c show the binary type, complementary pillar and groove topologies under investigation within the scope, where the width-to-period (w/Λ) ratio of gratings is optimized for tuning the effective refractive index (n_eff_) of the grating based composite layer, and the height/depth (h) of gratings is optimized for tuning the operation wavelength (λ_op_) in accordance with the QWIM. The placement period (Λ) of gratings is subwavelength in size; i.e., it is smaller than the operation wavelength (Λ ≪ λ_op_), so that gratings are ensured to enhance the transmission while eliminating diffraction and scattering mechanisms. This issue raises the cut-off wavelength (λ_c_) concept as a design parameter; i.e., the period should be chosen to be smaller than the cut-off limit (Λ ≪ λ_c_) in order to guarantee the SW nature of the gratings. In literature, the cut-off limit is mostly associated with the index of the substrate material and the period of the gratings, such as λ_c_ = Λ/n_si_^6,16–18^. Thus the upper limit for the period is determined as Λ < 2.3 μm by using the lower limit of the LWIR range as the cut-off, which is 8.0 µm. Although there is no theoretical lower bound for the period, it is still limited by the resolution limit of the lithography infrastructure, which is determined to be 1.0 μm for the designed configurations in this study. The other design parameters can also be roughly determined by using simple analytical equations in the literature, without resorting to any detailed analytical and numerical calculations^6^. Thus, the optimum n_eff_ is determined to be around 1.85, the depth/height to be around 1.35 μm, and the pillar width-to-period ratio (w/Λ) to be around 0.64. After obtaining these rough values, optimization ranges for the design parameters are determined by centring around these values, such as period sizes (Λ) with the values of 1.0 μm, 1.5 μm, and 2.0 μm; heights/depths (h) within a range from 0.8 μm to 1.8 μm, and pillar/groove w/Λ ratios within a range from 0.6 to 1.0. All variations correspond to a total of 19 different configurations, which allows a detailed analysis and verification of the proposed simulation technique and its findings. After determining the ranges, 2D optimizations over various depth/height values and various width-to-period ratios are performed through the proposed simulation technique, maximizing the average LWIR transmission for binary type pillars and grooves with square cross-section. The proposed simulation technique basically combines the efficient 3D complex geometry handling capability of the packaged programs with the efficient solution capability of Fresnel equations for electrically large problems. The reflection calculated through packaged programs is embedded into Fresnel equations to obtain the transmission through the patterned wafer, the details of which can be found in Methods section. Figure 2b,d show their LWIR optimization results, assuming a periodic placement of Λ = 1.0 µm. Through optimizations, it is observed that similar maximum average LWIR transmissions are obtained for both pillars and grooves, nearly 90% for the double side patterning and 64% for the single side patterning. Moreover, both the depth/height and the width-to-period ratios, which are optimized to 1.3 µm and 0.8 respectively, are also determined to be the same for both grating types. These optimum points are perturbed slightly with other periods under investigation, which are Λ = 1.5 µm and Λ = 2.0 µm. It is also observed that if the cross-section of the gratings is changed from square to circle, a similar maximum LWIR transmission around 90% with the same optimum height/depth of 1.3 µm is obtained. For the circular cross-section, the optimum grating width-to-period ratio, which is parametrized as diameter-to-period ratio (D/Λ) due to geometry, is determined to be 0.9. The difference between the optimum w/Λ and D/Λ is not surprising, considering the EMA since the volumetric ratio of the silicon becomes the same with these different values, which is found to be around 64%. These results show that the circular cross-section should not be preferred over the square one since the optimum D/Λ is closer to 1.0, which makes this type of gratings more sensitive to the fabrication related variations.

**Figure 3:**
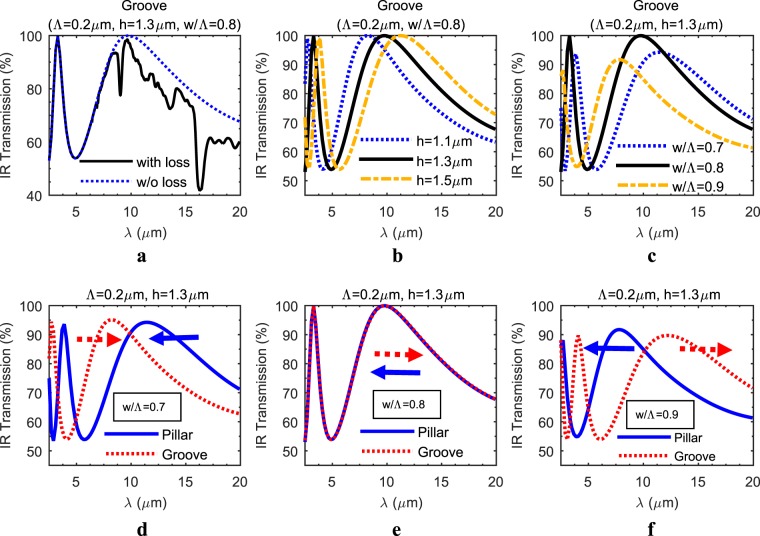
Simulation results for spectral IR transmission of double side patterned Si wafer. These simulations examine the effect of material loss (**a**), the variation in depth/height (h) (**b**) and width-to-period ratio (w/Λ) (**c**) for groove type SWARGs on the spectral IR transmission of the double side patterned silicon wafer, which are as predicted in accordance with the QWIM. It is also observed that as the w/Λ ratio is swept while the depth/height is kept constant around h = 1.3 µm, the spectral responses of the complementary pillar and groove structures shift in a symmetric way around a centre w/Λ ratio that is also the optimized point for the maximum average LWIR transmission (**d**–**f**). These results are obtained under true SW assumption (Λ = 0.2 µm).

The similarity observed within optimum design parameters for both types of SWARGs is analysed further over an extended LWIR range (2.5–20 µm), as opposed to previously obtained average LWIR results, in order to clarify the origin of this observation. Hence a spectral analysis over the extended LWIR range is performed with a period size lower than 0.7 μm, considering the previously defined cut-off limit expression (λ_c_ = Λ/n_si_) and the minimum operation wavelength of the extended LWIR range, which is 2.5 µm. However, it is observed that if the entire extended spectral response is monitored as the Λ is decreased, while w/Λ and h are set to optimum points and kept constant, the spectral response starts to converge to the same characteristics when λ_c_ < Λ/10 is satisfied, which is a stricter limit than the previously defined λ_c_ = Λ/n_si_. The convergence assumes an absolute root-mean-square (RMS) error less than 0.5% between the extended LWIR responses of Λ = 0.2 μm and Λ = 0.25 μm cases. The absolute RMS error is calculated by taking the RMS of the difference between the two spectral responses through the relevant spectral range without any normalization applied. This stricter condition is referred as the deep SW limit with the rest of this study and also employed through the first simulation trails of spectral transmission responses, which is summarized in Figure 3. While examining the extended spectral responses, the material loss is disregarded without loss of generality since the loss is too high considering the thickness of an 8-inch wafer (around 700 µm), and the loss is shadowing the characteristic behaviour of the spectral transmission. Figure 3a shows the shadowing effect of the loss on the characteristic spectral response of a groove type SWARG. Figure 3b shows that changing the depth/height of SWARGs shifts only the centre wavelength of the maximum transmission without affecting the magnitude of the maximum while w/Λ ratio is kept constant. On the other hand, Figure 3c shows that changing the w/Λ ratio both shifts the centre of the wavelength and changes the magnitude of the maximum simultaneously while h value is kept constant. A similar observation is also made for the pillar type SWARGs. These observations are as expected since the varying w/Λ ratio is directly affecting the effective refractive index of the composite structure and varying the height/depth is directly related to the quarter wavelength condition. Figure 3d–f proves that the entire extended spectral responses of pillars and grooves shift in adverse directions, i.e., from lower wavelength to higher or vice versa while the w/Λ ratio is swept around the optimum point, and the h value is kept constant, which can be attributed to the topological complementary nature and also to the subwavelength behaviour of binary pillar and groove type gratings. As the core result of this study, it is observed that these two spectral responses match exactly at a very specific w/Λ ratio of 0.8, which is also the optimum point for the maximum average LWIR transmission for both type SWARGs.

**Figure 4:**
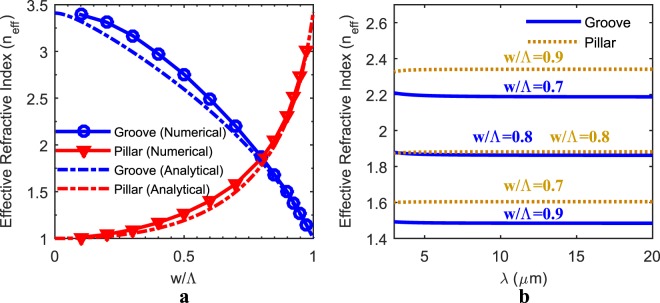
Effective indices of both grooves and pillars. The effective indices are calculated with changing w/Λ ratios at a single wavelength of 10 µm both numerically and analytically (**a**) and within extended LWIR spectral range numerically (**b**) under true SW assumption (Λ = 0.2 µm). Both numerical and analytical effective index extraction techniques confirm the matching spectral responses of grooves and pillars at a very specific width-to-period ratio of 0.8, where the n_eff_ is predicted to be around 1.85.

The matching spectral responses of complementary SWARGs is further investigated through the effective index extraction technique, which is mostly employed for metamaterials to monitor the optical behaviour of the composite layer^19^. It should be noted that these grating structures can also be considered as metamaterials since periods of the SWARGs are in SW regime. Figure 4a shows the effective refractive indices (n_eff_) calculated at an operation wavelength of λ_op_ = 10 µm with varying w/Λ ratios for both pillars and grooves, assuming a period of Λ = 0.2 µm. The crossing-over point at w/Λ = 0.8 and n_eff_ = 1.85 confirms the observation regarding the similar transmission responses for the complementary structures. At the crossing-over point, both pillars and grooves have the same w/Λ ratio, which suggests that they are structurally complementary. They also have the same n_eff_ around (n_si_)^0.5^ at this point, which suggests that they are optically indistinguishable, and present the optimum refractive index maximizing the transmission with the right choice of depth/height. These observations are also examined through an analytical approach to enhance the physical insight, which is based on a field-equivalent circuit model, i.e., the effective capacitance method, derived for the analysis of SW regime all-dielectric metamaterials (ADMs)^20^. Equation (1) shows the analytically derived formula for a normal incidence case, where an ADM composite material is created by embedding 2D array of square pillars of width w and of refractive index n_2_ into a continuous media of n_1_ with a placement period smaller than the operation wavelength (Λ << λ_op_). The complementary counterpart of this topology is obtained by replacing n_1_ with n_2_ and vice versa. Figure 4a also shows the effectiveness of the analytical model for the calculation of n_eff_, which is compared with the numerical extraction technique, as they both accurately predict the same crossing-over points.

1 $${n}_{eff}={[{n}_{1}^{2}(1-\frac{w}{{\rm{\Lambda }}})+\frac{{n}_{1}^{2}{n}_{2}^{2}}{{n}_{1}^{2}+{n}_{2}^{2}(\frac{{\rm{\Lambda }}}{w}-1)}]}^{1/2}$$

EMA suggests that while w/Λ ratio is swept from 0.0 to 1.0, the n_eff_ of a pillar should vary from n_air_ to n_si_ since the composite layer will evolve from air to silicon, and the n_eff_ of a groove should vary from n_si_ to n_air_ since it will evolve vice versa. Hence it is obvious that there should be a complementary case where both pillars and grooves have the same effective refractive index; i.e., they are optically indistinguishable. However, this approach does not explain for n_eff_ to be around n_si_^0.5^ = 1.85. On the other hand, if Equation (1) is examined, which is based on a field-equivalent approach, it suggests that when both grooves and pillars have the same n_eff_, they both are approximately index-matched to air (n_eff_ ≈ n_si_^0.5^) and are also structurally complementary, which explains for the observation for pillars and grooves turning out to be complementary when they are separately index-matched to air. Figure 4b shows the numerically extracted spectral variation of indices for both grooves and pillars with changing w/Λ ratios within the entire extended LWIR spectrum instead of a single wavelength. It is observed that grooves and pillars are optically indistinguishable through the entire extended LWIR range around w/Λ = 0.8, not just for one specific wavelength, which also explains for the spectral matching through the entire extended range.

### Simulation vs. Measurement

**Figure 5:**
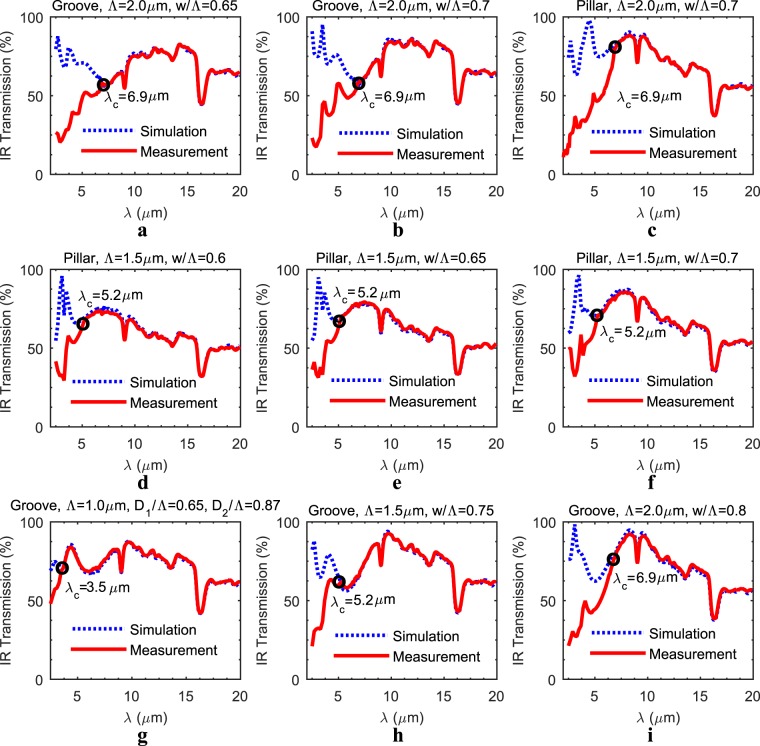
A comparison between the measured and the simulated IR transmission of both grooves and pillars. The comparison is done through various period sizes and various w/Λ ratios for both pillar and groove type SWARGs, assuming double side patterning (**a**–**i**). A good spectral agreement between the measured and the simulated data is observed for all cases within the SW regime with an absolute RMS error less than 5.4% The classical relation between the period size and the SW cut-off limit (λ_c_/Λ ≈ n_si_) is also observed (**g**,**h**,**i**).

IR transmission measurements are performed with the reflection/transmission (R/T) module of an IR-VASE ellipsometer. Figure 5a–i show a comparison between simulations and measurements for some of the configurations, which are changing in period sizes, width-to-period ratios, or SWARG types. For a fair comparison, the designed topological parameters are updated with the measured ones since it is observed that the designed and measured topological parameters might differ by a value of up to 23% for the depth/height and 17% for the w/Λ ratio. Throughout these comparisons, firstly the cut-off limits are observed for all configurations since fabricated period sizes are larger than the cut-off limit for the extended LWIR range, and since the proposed simulation technique is only valid within the SW regime. Figure 5g–i show that the cut-off limits are determined for various period sizes, such as λ_c_ = 6.9 µm for Λ = 2.0 µm, λ_c_ = 5.2 µm for Λ = 1.5 µm, and λ_c_ = 3.5 µm for Λ = 1.0 µm by spotting the data point where the simulations and the measurements start to diverge, all verifying the classical limit of λ_c_/Λ ≈ n_si_. Secondly, employing the actual period sizes proves to be more successful with the matching of simulations and measurements of spectral transmission responses especially nearby the cut-off when compared to the period sizes determined with the classical or deep SW cut-off limits. This is also an expected result since the transition nearby the cut-off, which is from SW regime (Λ << λ_op_) to near wavelength regime (Λ ≈ λ_op_), is also accounted for with the utilization of the actual period sizes. Lastly, an excellent spectral agreement between the measured and the simulated data is achieved for all configurations within the SW regime, as already mentioned previously, with an absolute root-mean-square (RMS) error less than 5.4% within the spectral range up to the cut-off wavelength.

**Figure 6:**
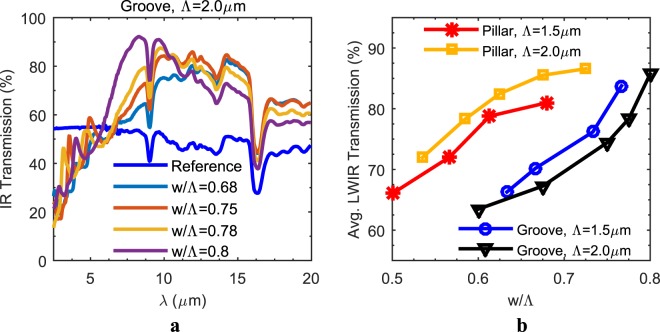
The measured spectral and average IR transmission for both pillars and grooves with changing w/Λ ratios. The gradual increase within the spectral IR transmission for a groove type SWARG with increasing w/Λ ratio for a period of Λ = 2.0 µm is observed experimentally (**a**). The increasing trend for the average LWIR transmission with the increasing w/Λ ratio up to 0.8 for both SWARG types with periods of 1.5 µm and 2.0 µm is proven experimentally (**b**), all assuming a double side patterning.

Figure 6a shows the measurement results proving the gradual increase within the extended LWIR range with increasing w/Λ ratios from 0.65 to 0.8, also including the reference measurement obtained before the patterning, for a groove type SWARG with a period size of 2.0 µm and a depth of 1.3 µm, which is as predicted by the simulations. A similar gradual increase is also observed for other configurations, and Figure 6b presents a refined representation of these results, i.e., the gradual increase within the average LWIR transmission with increasing w/Λ ratio for all configurations, which is also as predicted through simulations (see Figure 2b,d). The decreasing trend within the range from 0.8 to 1.0 with the increasing w/Λ ratio is not observed through measurements due to lithographic limitations. Nevertheless, it is safe to claim that 0.8 is the optimum w/Λ ratio maximizing the average LWIR transmission, considering the great agreement between simulations and the measurements. According to simulations, the increasing trend of average LWIR transmission with increasing w/Λ ratio up to 0.8 is expected to be different for pillars and grooves; however, it should be similar for the same type of gratings with different periods. Although there is a great resemblance between them, there is still a discrepancy, which is mainly due to fabrication errors and due to the fact that variations in depth/height of gratings are not included in Figure 6b. It is also observed that a maximum average LWIR transmission of 87% is achieved with double side patterning of the wafer, and the loss by 13% is mostly due to the material loss of Si, the narrow resonance characteristic of the QWIM technique, and partially due to fabrication errors.

**Figure 7:**
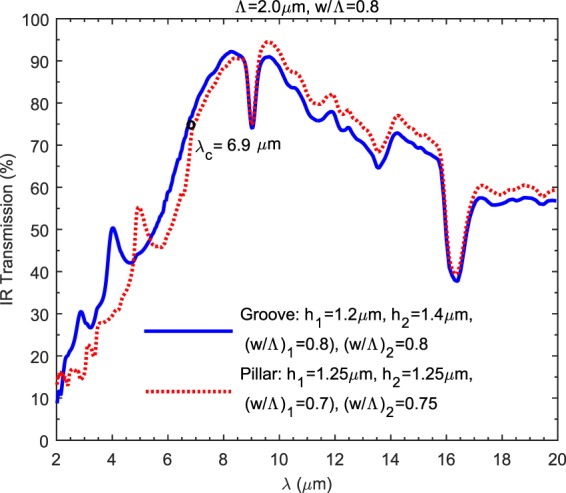
A comparison between the measured spectral IR transmission of complementary pillar and groove with a width-to-period ratio of w/Λ = 0.8 and for the same period of Λ = 2.0 µm. A spectral matching is observed with the measured transmission responses of both type SWARGs up to the cut-off wavelength (λ_c_ = 6.9 µm) as predicted by the simulations, which employs the optimum configurations for the maximum average LWIR transmission and assumes double side patterning. The minute discrepancy is mostly due to fabrication errors.

Finally, Figure 7 shows the matching transmission spectra for pillar and groove type SWARGs through measurements when the optimum topological configurations are employed, and the optimized structures are almost structurally complementary as the core result of this study. The minor discrepancy is due to the fabrication errors regarding the variations in height/depth, the width-to-period ratios observed on both sides of the wafer (h_1,_ h_2_, (w/Λ)_1_, (w/ Λ)_2_) and due to the fact that the actual period (Λ) is larger than the deep SW limit; i.e., the SW assumption is disturbed slightly. The gradual shift with varying w/Λ ratios for the entire spectral response of pillars and grooves (see Figure 3d–f) is not clearly observed since the material loss shadows this characteristic behaviour, which is also as predicted by the simulations.

## Discussion

A detailed spectral analysis is performed comparing the IR transmission of complementary, binary type pillars and grooves that are fabricated on an 8-inch DSP Si cap wafer with various topological configurations by using both simulations and measurements. A unique hybrid simulation technique is proposed for the analysis, which proves to be effective, considering the great agreement between the simulations and the measurements. Both simulations and measurements suggest that when optimum topological configurations for pillar and groove type SWARGs are examined to maximize the IR transmission through the silicon wafer, the optimum grating structures should present a similar spectral IR transmission and should be topologically complementary. This observation is also examined and confirmed through EMA and through an analytical model based on a field-equivalent circuit model. Thus, the same maximum average LWIR transmission is predicted to be around 90% with the simulations and verified to be around 87% with the measurements for both pillars and grooves employing the similar optimum topological parameters. Focusing on the optimum w/Λ ratio, which is found to be around 0.8, although the volumetric silicon-to-air ratio is different with the pillars and grooves, 64% and 36% respectively, both type SWARGs exhibit similar spectral IR transmission. It is also observed that the optimum design parameters obtained with the proposed method are not that different from the ones obtained through the rough analytical calculations. And it is verified that the deviation from the SW regime at the cut-off limit within the measured spectrum can be easily predicted by a simple equation as already highlighted in the literature, λ_c_/Λ ≈ n_si_. Finally, these findings are justified not only through measurements with an exhaustive testing executed with the above mentioned 19 different configurations but also through numerical simulations and an analytical model.

## Methods

### Simulation Technique

The proposed simulation technique is based on modifying the reflection (R) term of analytical, incoherent Fresnel equations for the normal transmission through a thick dielectric slab to account for the existence of gratings. In other words, the transmission through a double side patterned Si wafer is simplified into a transmission through a thick, plain dielectric slab with a modified surface reflection term. Equation (2) shows the simple formula that is used to calculate the reflection from an interface separating the air from the plain silicon wafer before the patterning. For the plain case, the transmission is obtained by embedding the Equation (2) into the Fresnel formula, shown in Equation (3). However, the existence of gratings prevents the utilization of this simple formula of Equation (2) since the Si surface is no longer plain. Thus, a modified version of the reflection term is needed to account for the gratings, which can be obtained through 3D numerical EM simulations with suitable boundary conditions (BCs) applied.

2 $$R={(\frac{n-1}{n+1})}^{2}$$

3 $$T=\frac{{(1-R)}^{2}{e}^{-\frac{4\pi kt}{\lambda }}}{1-{R}^{2}{e}^{-\frac{8\pi kt}{\lambda }}}$$

**Figure 8:**
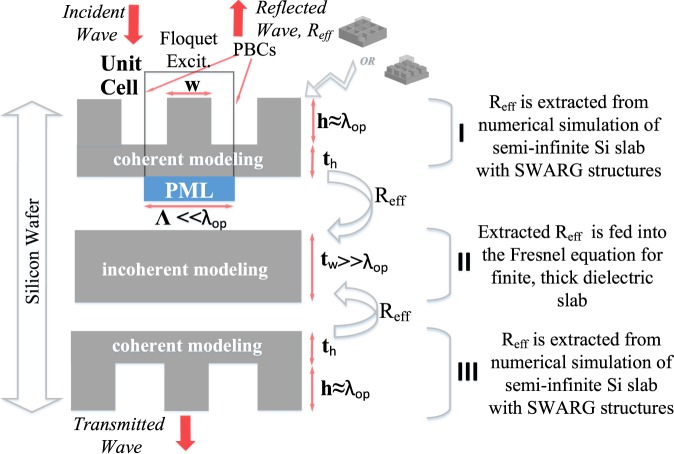
2D schematic of the structural decomposition of double side patterned silicon wafer through the proposed simulation technique. Since gratings have a depth around a few micrometres due to quarter wave condition, the region I and III containing these structures are modelled with coherent interaction, and the modified reflection data is extracted through numerical simulation programs, such as HFSS, Lumerical, etc. The region II containing the thicker Si part is modelled through incoherent Fresnel equation, which incorporates the extracted modified reflection data to account for the gratings.

Double side patterned silicon wafer is spatially decomposed into two shallow regions consisting of SWARGs and one thick wafer with modified reflection term embedded in accordance with the proposed simulation technique. Figure 8 presents the 2D schematic of the spatial decomposition, where the thick Si slab region (II, t_w_ ≫ λ_op_) is sandwiched between the two shallow regions (I and III, h ≈ λ_op_) composed of gratings. 3D numerical EM simulation setup is employed to extract the modified reflection term due to gratings in shallow regions. Within the scope of this study, HFSS is employed to form the 3D numerical EM setup, which is based on finite element method. The modified reflection term is embedded into the incoherent Fresnel equation to handle the EM interaction through the thick Si slab of region II. Figure 8 also shows the 2D schematic of the unit cell definition for gratings with a period of Λ and height/depth of h, which is employed through 3D simulations. The application of suitable BCs for the 3D EM modelling is vital for having an accurate and precise measure of the modified reflection data. Accordingly, periodic boundary conditions (PBCs) are used to decrease the simulation volume since these structures are placed in the form of arrays. A perfect matched layer (PML) BC is defined on the opposite side of the surface that consists of gratings to emulate a semi-infinite medium. The semi-infinite consideration is an important point with the proposed simulation technique, where the need is clarified as the reflection term of the Fresnel equation is examined carefully. Equation (2) suggests that the reflection (R) term corresponds to the reflection from a semi-infinite dielectric slab; and thus, the setup to extract the modified reflection data is also expected to mimic this semi-infinite scenario for the modified reflection to be utilized within the Fresnel equations directly. Another important point is the use of the handle thickness (t_h_) within the unit cell, which enables the quarter wave interaction to take place. The handle thickness is determined to be around 0.5 μm with a simple field based convergence test. Through the region II, the interaction of the EM wave with the thick Si wafer is handled by embedding the modified R term and the wafer thickness (t_w_) into the Fresnel equation. The EM interaction through the region II is modelled incoherently since the phase coherency is lost as the wave propagates throughout the thick wafer, which has a thickness of 720 µm for an 8-inch wafer in this scenario, and which justifies the need for incoherent Fresnel equations.

### Fabrication

**Figure 9:**
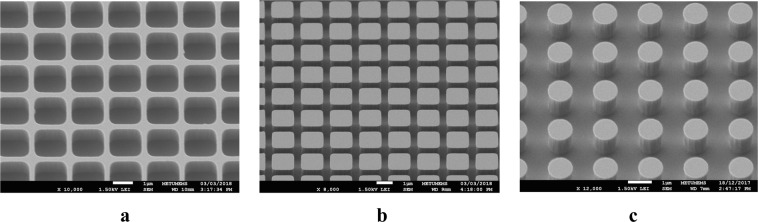
SEM images of various SWARG configurations patterned on the DSP Si wafer. Both grooves (**a**) and pillars (**b**,**c**) with various period sizes, cross-sectional profiles (circular or square-like), and w/Λ ratios are fabricated successfully by patterning through reactive ion etching (RIE) with a one-mask process.

Gratings are fabricated through a one-mask process with a reticle design where various configurations are placed with changing period sizes and width-to-period ratios. Both the dark field and clear field concepts are utilized simultaneously within the same reticle to be able to make a differential comparison between the grooves and pillars. For the process, first, the lithography is performed through an ultraviolet (UV) exposure on a photoresist (PR), which is deposited on the optical grade, DSP silicon wafer. After developing the PR, the wafer is exposed to the reactive ion etching (RIE) to engrave the pillars and grooves. Then the residual of the PR layer is stripped away, leaving the pillar and groove formations on one side of the wafer. The same procedure is repeated for the other side of the wafer; thus, the double side patterning is achieved. Figure 9 shows the scanning electron microscope (SEM) images for some of the fabricated pillar and groove type gratings. All configurations, 19 in total, are fabricated successfully with tolerable deviations from the designed ones regarding the dimensions.

### Measurement

IR measurements are performed with the reflection/transmission (R/T) module of J.A. Woollam IR Ellipsometer, with a resolution of 8 cm^−1^, a bandwidth of 0.04 μm, and scans/spectrum of 50, which are the settings of the measurement device.

## Data Availability

The datasets generated during and/or analysed during the current study are available from the corresponding author on reasonable request.
